# Diagnosis and indications for the extraction of third molars - The SECIB clinical practice guideline

**DOI:** 10.4317/medoral.26524

**Published:** 2024-02-18

**Authors:** Mª Ángeles Sánchez-Garcés, Jorge Toledano-Serrabona, Octavi Camps-Font, María Peñarrocha-Diago, Alba Sánchez-Torres, Gemma Sanmartí-Garcia, Erika Vegas-Bustamante, Rui Figueiredo, Eduard Valmaseda-Castellón, Cosme Gay-Escoda, On behalf of the Sociedad Española de Cirugía Bucal

**Affiliations:** 1 Faculty of Medicine and Health Sciences, University of Barcelona, Barcelona, Spain; 2 Faculty of Medicine and Dentistry, University of Valencia, Valencia, Spain

## Abstract

**Background:**

The removal of third molars (3Ms) is the most frequent surgical procedure in the field of Oral Surgery. As a result, the Spanish Society of Oral Surgery (SECIB) aims to create a Clinical Practice Guideline (CPG) that offers evidence-based recommendations for optimal clinical practice. Specifically, the CPG will focus on providing guidance regarding the indications and criteria for clinical and radiological diagnosis of patients with 3Ms.

**Material and Methods:**

This CPG was developed by the SECIB, following the methodological guidelines described in the methodological manual for the “Development of Clinical Practice Guidelines in the National Health System”. Several PICO questions related to the diagnosis and indications for the extraction of 3Ms were formulated. The leading experts carried out the evaluation of the evidence and the formulation of specific recommendations.

**Results:**

A total of 17 PICO questions were evaluated, addressing the indications, prognosis, diagnosis, and cost-benefit relationship of 3M extraction.

**Conclusions:**

The present Clinical Practice Guideline provides evidence-based recommendations on the diagnosis and indications for 3M extraction. These evidence-based recommendations can assist healthcare professionals and the general population in making informed decisions regarding the management of 3Ms.

## Introduction

The extraction of the third molar (3M) is the most common surgical intervention in the field of Oral Surgery. 3Ms that are erupted or impacted are extremely common in the general population and can be the cause of various complications. These complications include infection, periodontal pathology, caries in the second and third molars, root resorption, cysts, and associated tumors. In cases where there are symptoms or pathology, the indication for extraction is generally accepted (1) . However, there is controversy regarding the extraction of asymptomatic 3Ms that are free of pathology. This clinical decision-making depends on factors such as age, anatomical relationships, general health status, as well as patient preferences and lifestyle, among others (2,3).

Clinical Practice Guidelines (CPGs) provide protocols, algorithms, and recommendations based on available scientific evidence, aiming to assist both healthcare professionals in their daily practice and patients in making decisions about their health. The scope of this CPG is to address unanswered or partially resolved questions in other CPGs regarding the "Diagnosis and indications for the extraction of third molars” (4,5,6-13,14). However, the different issues and controversies surrounding pre and postoperative pharmacological guidelines and the surgical technique for the removal of 3Ms have not been addressed, as they deviate from the defined objectives.

Thus, the main aim of present Clinical Practice Guideline is to summarize evidence-based recommendations for the indications of third molar extractions, based on the best available evidence and/or expert knowledge.

Material and Method

- General framework

The CPG has followed the guidelines outlined in the document "Development of Clinical Practice Guidelines in the National Health System. Methodological Manual" (15) and the available recommendations in “Guía Salud” (https://portal.guiasalud.es) (16). This CPG represents an update of a previous version published by SECIB in the year 2017. The updating process was carried out in three consecutive phases, some of which ran concurrently at times.

Phase 1: Definition of the scope of the guideline and literature search.

Phase 2: Generation of documentation by the group of experts. For each clinical problem addressed, a self-developed working sheet was created. A total of 17 clinical questions were developed regarding the diagnosis and indications for the extraction of 3Ms.

Phase 3: Development and standardization of documents.

- Eligibility criteria and electronic search

For each clinical question formulated by the authors of this CPG, a PICO question (Patient - or target population to which the intervention is directed -, Intervention/Comparison - intervention measured in comparison or not with another that is performed -, and Outcome - expected outcome when applying the indicated intervention) was developed to establish the inclusion/exclusion criteria for the studies. Articles that were not written in English, French, or Spanish were excluded. Finally, the article selection process was limited to the last 10 years. A screening of titles and abstracts of the articles identified in the searches was conducted to eliminate those that clearly did not meet the search criteria.

The available scientific literature was reviewed up until January 2023. For each question developed in this CPG, a specific search strategy was designed. In Annex 2 of the 3M Clinical Practice Guideline (available at https://portal.guiasalud.es/gpc/diagnostico-e-indicaciones-para-la-extraccion-de-los-terceros-molares-actualizacion-2023/), readers can find the references used during the current upgrade. The electronic databases Pubmed/Medline and EMBASE were employed for the literature search.

- Evaluation and synthesis of the evidence

Following the initial screening, the selected articles underwent a comprehensive evaluation using checklists provided by the Scottish Intercollegiate Guidelines Network (SIGN). These checklists were applied to assess randomized clinical trials (RCTs), systematic reviews and meta-analyses, cohort studies, case-control studies, diagnostic test studies, and economic evaluations. Additionally, OSTEBA forms were used to evaluate case series, and the AGREE tool was employed for assessing CPGs.

- Development of recommendations

After categorizing each of the evidence found, which corresponds to a PICO question, we assign a grade of recommendation, classified as letters A, B, C, or D, with the following meanings:

A: Based on at least one meta-analysis, systematic review, 1++ clinical trial, or a body of scientific evidence composed of studies classified as 1+ and showing substantial consistency among them.

B: Based on a body of scientific evidence composed of studies classified as 2++, directly applicable to the target population of the guideline and demonstrating substantial consistency among them; or extrapolated from scientific evidence from studies classified as 1++ or 1+.

C: Based on a body of scientific evidence composed of studies classified as 2+, directly applicable to the target population of the guideline and demonstrating substantial consistency among them; or extrapolated from scientific evidence from studies classified as 2++.

D: Based on level 3 or 4 scientific evidence, or extrapolated from scientific evidence from studies classified as 2+.

## Results

- Question 1: Which patient populations with associated pathologies (such as pericoronitis, cysts, caries on the distal aspect of the second molar (2M), periodontal pathology of the 2M, mandibular fracture, etc.) achieve a better clinical outcome (with fewer complications) through extraction or by adopting a conservative therapeutic approach (clinical and radiological monitoring)?

After conducting a literature review, a total of seven articles (Annex 2 of 3M CPG) were selected. The consulted papers exhibited a lack of methodological quality and scientific consistency. Furthermore, the presence of numerous confounding factors complicates the formulation of definitive conclusions. It can be concluded that (Grade of recommendation D):

Due to the well-documented increase in morbidity associated with impacted/included 3Ms (non-restorable caries, fractures, infection, periodontal pathology, recurrent pericoronitis, cysts, and tumors), if pathology appears, extraction is indicated.

In cases where there is no infection or other associated pathology, extraction is not indicated.

The extraction of a 3M with signs and/or symptoms of periodontal disease improves the periodontal health of the second molar.

The postoperative quality of life of patients with symptomatic 3Ms and pathology improves after surgical extraction.

- Question 2: Do patients with an impacted third molar and high root development experience different postoperative morbidity compared to those with lower root development?

After the literature review, a total of five articles (Annex 2 of 3M CPG) were selected.

These articles exhibited a lack of methodological quality and scientific consistency. Thus, it can be concluded that (Grade of recommendation D):

The degree of root development is not associated with a higher risk of postoperative complications, nor does it influence the recovery period. The only complication that is increased with higher degree of root development is injury to the IAN.

The authors consider that the frequency of complications when extracting 3Ms with closed apices is higher compared to extractions when root development is incomplete (open apices), and clearly impact the postoperative evolution and quality of life.

Upper 3Ms with complete root development and those with less than half root development are associated with a higher frequency of postoperative maxillary sinus communications.

- Question 3: Are there preoperative clinical and radiological criteria related to the degree of surgical difficulty in patients with an indication for third molar extraction (short operative time and low morbidity)?

After the literature review, 10 articles were selected (Annex 2 of 3M CPG) to address this prognostic question:

Since there is evidence about the relationship of some radiological factors in estimating the degree of surgical difficulty, it is necessary to perform at least a panoramic radiograph on all patients to carefully evaluate the morphology of the 3Ms and their position relative to neighboring anatomical structures. The main radiological variables most related to surgical difficulty have been the position of the 3Ms, depth of dental impaction, relationship with the second molars, and the number, morphology, and curvature of the roots. Grade of recommendation A.

The postoperative course is worse with higher surgical difficulty. Likewise, the complication rate is higher in moderate and high 3Ms. Grade of recommendation A.

Demographic variables related to a higher difficulty in 3M extraction are patient age and a high body mass index (overweight). Grade of recommendation B.

A maximum mouth opening of less than 45 mm hinders 3M extraction surgery. Grade of recommendation B.

There is controversy regarding whether the level of clinical experience influences the assessment of surgical difficulty. It appears that both residents and experienced surgeons can assess the surgical difficulty of 3M extraction, but the surgical procedure duration is significantly shorter for experienced surgeons. Grade of recommendation A.

- Question 4: Do patients with a periodontal probing depth of 4 mm or more distal to the second molar (2M), whether they have undergone third molar extraction or not, have a higher incidence of generalized periodontal disease compared to those with a periodontal probing depth of less than 4 mm?

After the literature review, 18 relevant articles were selected (Annex 2 of 3M CPG):

There is insufficient evidence to determine that a periodontal probing depth ≥4 mm in the distal aspect of 3Ms is associated with a higher incidence of generalized periodontitis.

The deterioration of the periodontal condition in the area of the 3Ms should be considered as a criterion for extraction. Generally, following the extraction of impacted 3Ms, the periodontal parameters on the distal side of the 2Ms remain unchanged or even improve in many cases. Grade of recommendation A.

- Question 5: Does the extraction of 3Ms provide greater benefits in resolving anterior dental crowding (with or without orthodontic treatment) compared to a conservative therapeutic approach?

After the literature review, 12 relevant articles were selected (Annex 2 of 3M CPG).

The extraction of 3Ms to prevent, limit, or resolve the degree of anteroinferior dental crowding is not justified, as the available evidence indicates that there is no cause-effect relationship. Grade of recommendation C.

- Question 6: In patients without anterior dental crowding, does the extraction of 3Ms contribute to the maintenance of alignment in the anteroinferior teeth?

After the literature review, 5 relevant articles were selected (Annex 2 of 3M CPG).

There is no causal relationship between post-orthodontic treatment crowding and the presence of 3Ms. Grade of recommendation B.

The extraction of 3Ms is not justified as a preventive measure for the relapse of malocclusion in the anteroinferior sector after completing orthodontic treatment. Grade of recommendation B.

- Question 7: Do patients with a dental prosthesis who undergo third molar extraction experience higher associated morbidity compared to those who do not undergo extraction?

After the literature review, 6 relevant articles were selected (Annex 2 of 3M CPG). There is insufficient data in the scientific literature to make a recommendation regarding the morbidity associated with the extraction of a 3M or therapeutic abstention in patients with dental prostheses.

- Question 8: In patients undergoing third molar extraction, what risk factors influence their postoperative quality of life?

After the literature review, 2 relevant articles were selected (Annex 2 of 3M CPG) and the following key findings were reported:

Demographic factors, such as being over 21 years old and female gender, prolong the time needed for the recovery of quality of life after the extraction of 3Ms. Grade of recommendation C.

There is not enough scientific evidence to determine whether other factors may influence postoperative quality of life after the removal of 3Ms.

- Question 9: Do patients with asymptomatic 3Ms benefit from their extraction compared to non-extraction?

After the literature review, 22 relevant articles were selected (Annex 2 of 3M CPG). Authors found that there is clinical evidence supporting therapeutic abstention in cases of asymptomatic and pathology-free partially or totally impacted 3Ms. Grade of recommendation B.

- Question 10: Is age (more or less than 25 years) a factor related to the occurrence of morbidity associated with the extraction of the third molar?

After the literature review, 15 relevant articles were selected (Annex 2 of 3M CPG) and the following key findings were identified:

Considering the well-documented increase in morbidity associated with impacted 3Ms that correlates with patients' age (e.g., caries, periodontal issues, root resorption, etc.), it is advisable for patients with impacted 3Ms to undergo lifelong active surveillance. In the event of any pathology, extraction should be recommended at the earliest appropriate age for each case. Best clinical practices.

Given that the majority of the consulted studies associate the surgical removal of the 3M with an increased morbidity, which rises with the patient's age, the risk factors for extraction should be carefully evaluated, especially in cases of deep impactions. Grade of recommendation: C.Final del formulario

In cases where there is no infection or other associated pathology, extraction is not recommended at an advanced age. Best clinical practices.

- Question 11: Do patients with fully impacted 3Ms benefit from extraction? What are the guidelines to follow in patients with fully impacted 3Ms where extraction is not performed to avoid complications?

While several publications have addressed the extraction of asymptomatic and pathology-free 3Ms, none of them provide specific guidance on the management of asymptomatic mandibular 3Ms with deep impaction. Furthermore, the scientific literature analyzed has not led to an elevation in the recommendation grades formulated in Question 11.

After the literature review, 15 relevant articles were selected (Annex 2 of 3M CPG), and the following key findings were identified:

In asymptomatic, fully impacted 3Ms, extraction should only be considered in the presence of signs or symptoms of pathology. Grade of recommendation B.

The decision to extract an asymptomatic, fully impacted 3M should be assessed on a case-by-case basis, considering the risks, benefits, and the patient's own opinion. Grade of recommendation B.

There is no indication for prophylactic extraction of 3Ms in cases of patients at risk of mandibular fractures (e.g., contact or high-risk sports) because, although the presence of an impacted 3M favors fractures of the mandibular angle, its absence favors fractures of the mandibular condyle, which are much more complex to treat. Grade of recommendation C.

Asymptomatic, fully impacted 3Ms left in place should undergo lifelong active surveillance to detect potential pathologies. Grade of recommendation A.

Monitoring these cases should include a review of the patient's medical history, a thorough clinical examination, and radiological assessment performed by a clinician competent in the evaluation and treatment of 3Ms. Grade of recommendation D.

For patients in whom therapeutic abstention is recommended, it is advisable to undergo active surveillance with clinical check-ups every six months to one year, aligning with routine dental visits, and radiological monitoring through panoramic X-rays every two years. Best clinical practices.

- Question 12: In patients with an impacted third molar, when is the computed tomography (CT) recommended to prevent clinical and/or surgical complications?

After the literature review, 28 relevant articles were selected (Annex 2 of 3M CPG), and the following key findings were identified:

CT is not routinely recommended for the removal of impacted 3M. Grade of recommendation A.

The use of CBCT does not seem to reduce the risk of IAN injuries when compared to relying solely on panoramic radiographs for diagnosis. Nevertheless, CBCT proves beneficial for assessing the relationships between impacted 3M and the IAN canal, potentially aiding in surgical indication and planning. Grade of recommendation: B.

CT is not routinely indicated for cases of coronectomy of impacted 3Ms. Grade of recommendation C.

- Question 13: In which patients can the position of the third molar be related to the possibility of future clinical symptoms or the presence of pathology compared to those who remain asymptomatic?

After the literature review, 34 relevant articles were selected (Annex 2 of 3M CPG), and the following key findings were identified:

3Ms in vertical or distoangular positions, partially covered by soft tissues and close to the occlusal plane, have a higher risk of developing pericoronitis, indicating the need for prophylactic extraction. Grade of recommendation C.

In cases of horizontally or severely mesioangular positioned 3Ms in patients between 25-30 years of age, prophylactic extraction is indicated to prevent periodontal damage on the distal aspect of the second molars and the post-surgical sequelae that may occur if extracted after this age. Grade of recommendation B.

Prophylactic extraction is indicated for partially or fully erupted 3Ms in mesioangular or horizontal positions due to the higher frequency of distal caries on the 2Ms. Grade of recommendation C.

Prophylactic extraction of impacted 3Ms is not recommended in patients with a high risk of mandibular fractures (e.g., contact sports) despite the presence of 3Ms increasing the risk of mandibular angle fractures, as their absence increases the risk of mandibular condyle fractures. Grade of recommendation C.

- Question 14: In patients at high risk of intraoperative injury to the inferior alveolar nerve, does coronectomy have lower morbidity compared to complete extraction of the third molar?

After the literature review, 32 relevant articles were selected (Annex 2 of 3M CPG), and the following key findings were identified:

In cases of mandibular 3Ms identified preoperatively as having a high risk of IAN injury, the coronectomy technique (complete removal of the crown of the 3M while intentionally leaving the root in place) reduces the incidence of such injuries. Grade of recommendation B.

Performing a coronectomy does not significantly increase the risk of postoperative complications such as dry socket, postoperative infections, or pain compared to complete extraction of a mandibular 3M. Grade of recommendation B.

It is common to observe coronal migration of the remaining root of the 3M after performing a coronectomy, especially during the first year and in young patients. This migration may necessitate the extraction of the root in a small percentage of cases. Therefore, regular clinical and radiological follow-up of the patients is recommended. Grade of recommendation B.

- Question 15: What is the variation in economic costs associated with the extraction of the third molar depending on the level of patient care (primary care versus hospital care)?

After the literature review, 6 relevant articles were selected (Annex 2 of 3M CPG). The increased overall costs in the hospital environment are primarily attributed to the larger healthcare staff presence. Additionally, surgical interventions often involve intravenous sedation or general anesthesia, which requires patient transport to the hospital, typically located at a greater distance from the Primary Care Center. Grade of recommendation C.

- Question 16: What variation in economic costs does the extraction of 3rd molars (wisdom teeth) have in relation to the professional's training (generalist or specialized)?

After the literature review, 5 relevant articles were selected (Annex 2 of 3M CPG), and insufficient articles were available to provide conclusive findings regarding the PICO 16 question.

- Question 17: What is the cost-benefit variation of prophylactic extraction of 3Ms versus therapeutic abstention based on regular clinical and radiographic monitoring?

After the literature review, 13 relevant articles were selected (Annex 2 of 3M CPG) , and the following key findings were identified:

In the short and medium term, therapeutic abstention with clinical and radiographic monitoring proves superior to prophylactic extraction. This approach prevents an unwarranted intervention that may lead to transient discomfort, a brief period of incapacitation, intraoperative or postoperative complications, and avoidable expenditures. Grade of recommendation B.

However, prophylactic extraction should be considered based on an individual's risk of pathology and/or symptoms during the follow-up period, or the need for medical treatment that may subsequently contraindicate the procedure or increase the risk of complications. This decision should be reevaluated at each visit during clinical and radiological follow-up. Grade of recommendation A.

It is crucial to assess the individual risk of developing pathology. Patients with a higher likelihood of developing pericoronaritis, periodontal disease, and caries should undergo prophylactic extraction, as it is the most cost-effective option in these cases. Grade of recommendation B.

## Discussion

The present Clinical Practice Guideline is an update of the one published in 2017. This updated version has incorporated some additional evidence compared to its previous edition. Regarding associated pathology, the recommendation for extraction (Q1) remains unchanged. No new evidence has been presented concerning morbidity associated with the degree of root development (Q2). However, two new risk factors, overweight and reduced mouth opening, have been added to the clinical criteria related to increased surgical difficulty (Q3). No additional recommendations have been made concerning the relationship between generalized periodontal disease and increased probing depth in the second molars (Q4), the association between lower dental crowding or its prevention in relation to 3Ms (Q5, Q6), or the increased morbidity in cases involving patients who wear prostheses and have an impacted 3M (Q7).

The level of evidence has been maintained regarding the risk factors that influence the quality of life during the postoperative period (Q8) and the indication for extraction when the 3rd molars are asymptomatic and free of pathology (Q9). Age has been introduced as both a local and general risk factor. In the case of partially or fully erupted 3rd molars, which are typically extracted when they present complications (such as caries or local periodontal pathology) or due to the increased risk of tuberosity or jaw fracture following surgical extractions of 3Ms with advancing age, prophylactic extraction may be recommended (Q10).

Regarding the indication for a fully impacted 3M, this should be considered on a case-by-case basis, taking into account the patient's opinion and lifestyle, with active monitoring being mandatory when deciding on abstention (Q11). The Grade of recommendations for CBCT to prevent surgical complications have been changed, as it does not provide additional benefits compared to panoramic radiographs in cases where there is no suspected close relationship with the IAN or when planning coronectomy (Q12). The grade of recommendation regarding the abstention from surgical extraction of impacted 3Ms in patients at high risk of mandibular fracture has been raised by one level (Q13). Despite an increase in published studies on coronectomy, its indications and Grade of recommendation have not been modified (Q14).

After the guideline update, the Grade of recommendations regarding cost-benefit studies related to the location where extractions are performed (primary care/hospital), the professional category performing them, and prophylactic extraction versus active monitoring have not been changed (Q15, Q16, Q17).

Due to the extensive bibliography consulted to prepare this clinical practice guideline, a greater number of controlled randomized clinical trials with sufficiently representative sample sizes are needed to address some aspects that still generate controversy today.

In conclusion, this updated clinical practice guideline on 3Ms is a valuable document that aids in diagnosis and therapeutic indications based on different clinical scenarios, identifying which patients may benefit from surgical extraction and when it is unnecessary.
